# Visual Search as a Tool for a Quick and Reliable Assessment of Cognitive Functions in Patients with Multiple Sclerosis

**DOI:** 10.1371/journal.pone.0081531

**Published:** 2013-11-25

**Authors:** Kathrin S. Utz, Thomas M. A. Hankeln, Lena Jung, Alexandra Lämmer, Anne Waschbisch, De-Hyung Lee, Ralf A. Linker, Thomas Schenk

**Affiliations:** Department of Neurology, Friedrich-Alexander University Erlangen, Nuremberg, Germany; University Of São Paulo, Brazil

## Abstract

**Background:**

Despite the high frequency of cognitive impairment in multiple sclerosis, its assessment has not gained entrance into clinical routine yet, due to lack of time-saving and suitable tests for patients with multiple sclerosis.

**Objective:**

The aim of the study was to compare the paradigm of visual search with neuropsychological standard tests, in order to identify the test that discriminates best between patients with multiple sclerosis and healthy individuals concerning cognitive functions, without being susceptible to practice effects.

**Methods:**

Patients with relapsing remitting multiple sclerosis (*n* = 38) and age-and gender-matched healthy individuals (*n* = 40) were tested with common neuropsychological tests and a computer-based visual search task, whereby a target stimulus has to be detected amongst distracting stimuli on a touch screen. Twenty-eight of the healthy individuals were re-tested in order to determine potential practice effects.

**Results:**

Mean reaction time reflecting visual attention and movement time indicating motor execution in the visual search task discriminated best between healthy individuals and patients with multiple sclerosis, without practice effects.

**Conclusions:**

Visual search is a promising instrument for the assessment of cognitive functions and potentially cognitive changes in patients with multiple sclerosis thanks to its good discriminatory power and insusceptibility to practice effects.

## Introduction

With a prevalence of 40 to 65%[1,2], cognitive impairment is one of the most common symptoms of multiple sclerosis (MS) that may already arise at an early stage of the disease[3]. Typically affected domains are memory, attention, information processing and executive functions, whereas language and simple attention span are scarcely impaired (for review: 4-6). 

Notably, cognitive deficits negatively affect patients’ quality of life[7], ability to work[8] and rehabilitation outcome[9]. Despite those debilitating consequences of cognitive impairment, it is not routinely assessed during clinical examination or trials. One reason for this shortcoming is that most available cognitive tests have been designed for other patient groups (i.e. stroke) and thus are not sensitive enough to assess cognitive functions in MS, where, particularly during an early stage, only mild deficits predominate. Moreover, to cover the variety of potentially affected functions in MS the conduction of comprehensive test batteries is required, making assessment time-consuming and impracticable. Short screening tests (i.e. Paced Auditory Serial Addition Test; PASAT) are susceptible to measurement errors, assess only few facets of cognitive functioning and are not suitable to assess changes in cognitive functions due to considerable practice effects[10]. Self-reports are just as little suited for the assessment of cognitive deficits, since they strongly correlate with depressive symptoms[4] and diverge with objective measures of cognitive impairment[6].

**Figure 1:**
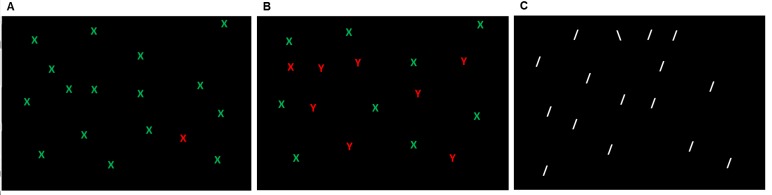
Illustration of the visual search tasks. A: single feature search – colour. The red X has to be found amongst several green Xs varying in number. B: feature conjunction search. The red X has to be found amongst green Xs and red Ys varying in number. C: difficult feature search – orientation. A white backslash (\) has to be found amongst white slashes (/) varying in number.

The importance of the assessment of cognitive impairment in MS patients on the one hand and the lack of suitable instruments for this purpose on the other hand, emphasize the need to identify a suitable measure of cognitive deficits in MS patients circumventing the problems of currently available tests. Such a test could be the visual search task. This paradigm was introduced by Treisman and Gelade[11] and is one of the best understood tasks in the field of cognitive neurosciences. In this task, a target stimulus, presented on a computer screen, has to be detected amongst similar distracting stimuli via button press (see Figure 1). 

Depending on the displayed array of stimuli visual attention or purely perceptual processes (figure ground segregation) are required[11]. This allows the separate assessment of sensory and attention-related deficits. Additionally, position priming can be induced by repeating the target location leading to faster responses, which is an indicator of implicit perceptual memory[12]. Apart from priming, practice effects are typically not observed[13], suggesting that the visual search task can be reliably used to assess changes in cognitive functions via repeated testing. To broaden the diagnostic information of the visual search task, the response key can be replaced by a touch screen, requiring the subject to directly touch the target on the screen[14]. In this way, indicators of visuomotor functions can be assessed such as movement time or accuracy of pointing. Moreover, using a pointing response instead of the more conventionally employed button-press response offers one further advantage. Conventionally the participant has to decide whether a target item is present or not and press the corresponding button to indicate their decision. However, this means that a substantial number of trials without a target item (catch trials) have to be presented. These trials are frequently not used for the final analysis. When a pointing response is used, the accuracy of the pointing response is proof that the target item has been detected. Catch trials are thus superfluous and thus more data can be collected in less time. 

To sum up, the visual search task seems to be an ideal tool for the assessment of cognitive impairment in MS: It assesses different facets of cognitive functions and motor behaviour, shows no practice effects and thus in principle could be able to assess disease- or medication-related changes in cognitive functions of MS patients.

In the present study we examined MS patients and healthy individuals both with our modified visual search task and with a set of standard neuropsychological tests. Our aim was to determine which cognitive assessment procedure would provide the most reliable discrimination between individuals with and without MS. 

## Materials and Methods

### Ethics Statement

The protocol was conducted in accordance with the Declaration of Helsinki II and was approved by the ethics committee of the Friedrich-Alexander University Erlangen-Nuremberg. All participants gave their written informed consent prior to the investigation.

### Participants

Thirty-eight outpatients (11 males, 27 females) with relapsing remitting MS according to the 2005 revised McDonald criteria[15] with a mean age of 36 years (standard deviation SD: 10.57; range: 21-60) were consecutively recruited. All patients were eligible for escalation therapy, meaning that they had experienced a relapse during medication and had shown signs of relapse as demonstrated by MRI in the preceding year, but had not experienced a relapse within 30 days prior to the examination, which was an exclusion criteria. Furthermore, patients were excluded in case of drug or alcohol abuse, diagnosed dementia or depression, an Expanded Disability Status Scale (EDSS)[16] score above 5.5, high-dose steroid therapy, or whenever another immune disease requiring immune suppression was diagnosed. 

The mean EDSS score of this group was 3.12 (SD: 0.98; range: 1-5) and the mean time since diagnosis was 95.59 months (SD: 80.42 range: 7-300). Fourteen patients had a functional deficit of the right arm (paresis, ataxia) and the mean visual function score within the EDSS was 0.76 (SD: 1.07; range: 0-4). MS medication taken during the last six months was distributed among patients as follows: None: 8; interferon beta 1a: 11; interferon beta 1b: 2; natalizumab: 8; glatirameracetat: 11; azathioprine: 1; fingolimod: 1. 

Additionally, 40 age- and gender-matched healthy individuals (12 males, 28 females) without neurological or psychiatric disorders with a mean age of 36.3 years (SD: 11.39; range: 23-56) were included in the study. The groups did not differ significantly regarding age (*t* (76) = 0.079; *p*=.973) or gender (χ^2^(1) = 0.01; *p*=.919). 

### Neuropsychological standard tests

A selection of seven neuropsychological tests was used as our baseline measurement. We selected tests which had been used before in MS research or formed part of a cognitive test battery for MS patients and focussed on those cognitive deficits which have been most commonly found in MS patients, namely deficits of attention and memory (see Table 1).

**Table 1:** Summary of the used neuropsychological standard tests.

Test	Cognitive function	Description
Brief Repeatable Battery of Neuropsychological Tests (BRB-N)[30]		
10/36 Spatial Recall Test (SPART; modified version)	Visuospatial learning and delayed recall	In our modified paper-and-pencil version the original pattern was presented on a sheet of paper (ten circles in a 6x6 grid pattern) for 10 seconds. The participants’ task was to replicate the spatial pattern as precisely as possible. They did this by drawing circles into the corresponding cells of an empty grid pattern on another sheet of paper. This procedure was repeated twice. After 15 minute delay (delayed condition) participants were asked again to reproduce the spatial pattern from memory, but this time the spatial pattern had not been shown again. In case more than 10 target circles were produced, the number of excess circles was subtracted from the number of correctly assigned circles. For the immediate condition all three trials were combined yielding a maximal score of 30; for the delayed condition a maximum score of 10 could be achieved
Paced Auditory Serial Addition Test 3’’(PASAT[30,31])	Auditory information processing speed and selective attention[30]	Sixty-one numbers, orally presented by tape every 3 seconds (PASAT-3ˡˡ) have to be added such that the just heard number has to be summed up to the previously presented number. The total of correctly added pairs of numbers is counted (maximal 60)
Wechsler Memory Scale Revised Edition (WMS-R[32]); German edition[33]		
Digit span forward/backward	Verbal short-term memory	Sequences of digits increasing in number are orally presented and the subject has to either repeat it in the original (forward) or the reversed order (backward). The number of correctly reproduced sequences is scored, separately for the forward and backward trials
Spatial span backward/forward	Visuospatial short-term memory	Eight blocks on a board are presented. The experimenter taps a certain sequence of blocks and the subject has to immediately reproduce the sequence in forward or backward order. The number of correctly reproduced sequences is scored, separately for the forward and backward trials
Logical memory I (modified)	Verbal long-term memory	For this test we used a simplified version, i.e. only one of the two stories was used and one point was given whenever the item was recalled with its precise wording, half a point was awarded, when the correct meaning was recalled, but the wording differed. To ensure that our scores were comparable to those obtained with the original version of this test, we multiplied the final score by a factor of 2. Thus the maximum score in our version was identical to that of the original version, namely: 50
Testbatterie zur Aufmerksamkeitsprüfung (TAP[34]): computerized German test battery		
Go/Nogo (version ‘two out of five’)	Selective attention	A series of five squares filled with different patterns are presented in random order on a computer screen. Two of them are critical target stimuli that require a button press as quickly as possible, whereas no response is needed to the other three stimuli. Reaction time and the number of errors are scored
Divided attention (version I/auditive-visual)	Divided attention	A quadratic array of 4x4 dots is displayed. Between six and eight Xs appear on the locations of the different dots. The participant is required to press a button as quickly as possible, whenever four of these Xs constitute a square. Simultaneously, a high-pitched and a deep tone are presented in alternate order. Whenever the same tone appears in succession, the participant has to press a button as quickly as possible. The number of omissions is scored

### Visual search task

**Figure 2:**
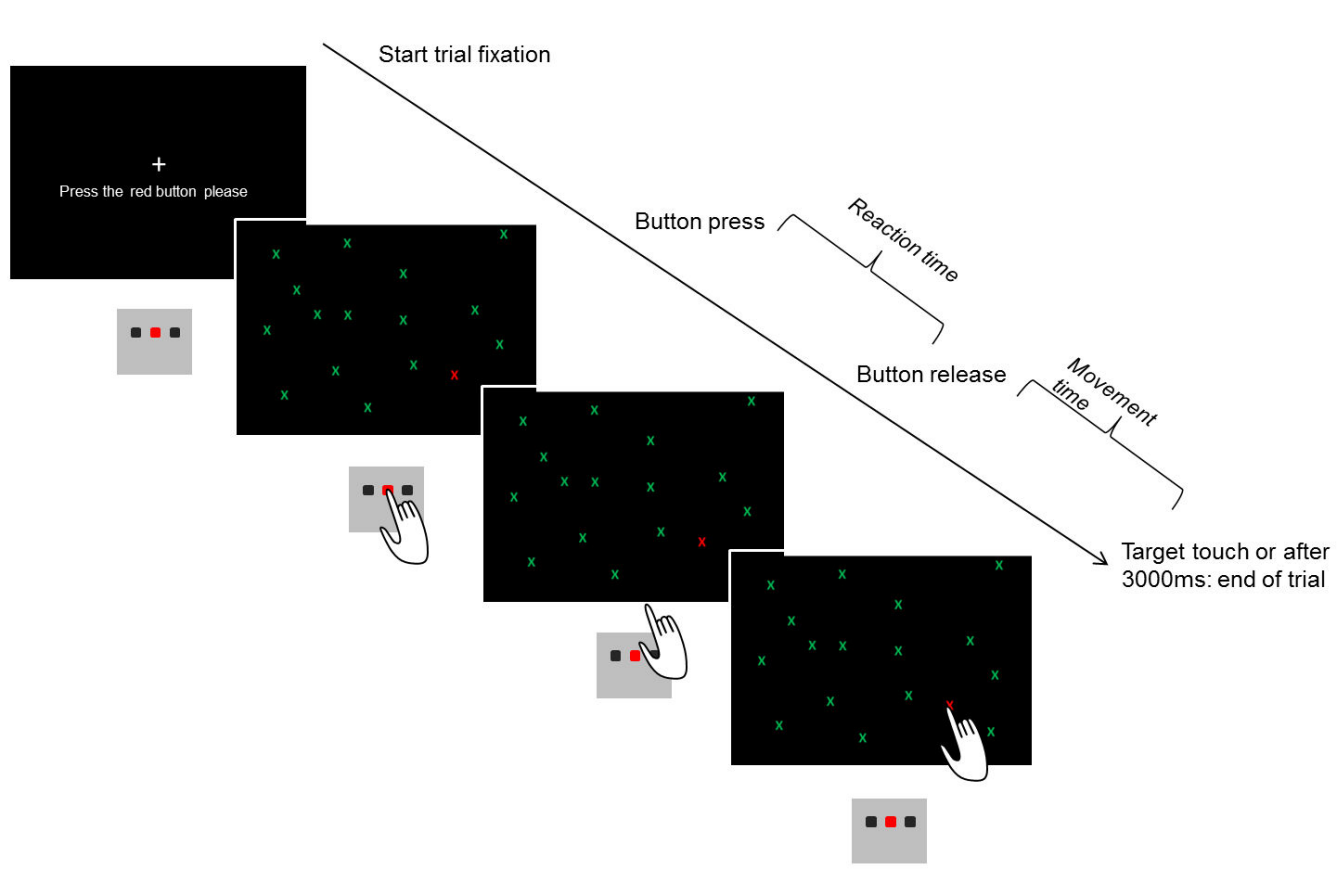
The sequence of a trial using the example of the single feature search. At the beginning, a central fixation cross was displayed along with the instruction to hold down the start button. Participants had to press a button on a Serial Response Box (Psychology Software Tools, Inc., Pittsburgh, PA). At the moment of button press, the stimuli appeared on the screen. The participants were instructed to visually search the target, and release the button only after they had detected the target. Next they should touch the target on the screen. The trial was completed (and the next trial initiated) when the touch screen detected a touch or when more than 3000 milliseconds (ms) had elapsed since button release. Trials, that were aborted because no touch was registered within the 3000 ms time window, were excluded from the analysis.

In this task, participants had to search a target amongst a varying number of distractors. Three different versions of visual search tasks were conducted (see Figure 1). All tasks were programmed in E-prime 2 (Psychology Software Tools, Inc., Pittsburgh, PA). All stimuli were presented against a black background on a 17” computer monitor covered by an add-on touch screen (KTMT1700 USB; Magic touch, Keytec Inc., Garland, Texas) and driven by a Pentium M 750 notebook. Table 2 shows the different parameters of the search tasks, and Figure 2 illustrates the sequence of each trial.

**Table 2:** Parameters of the different visual search tasks.

Block no.	Task	No. of practice trials	Set sizes: green X	Set sizes: red Y	Set sizes: white /	No. of trials per set size/set size combinations*	No. of standard/priming trials	Total no. of experimental trials
1	single feature search – colour	10	7/15/31	–	–	12	18/18	36
2	feature conjunction search	10	3/4/7/8/15/16*	3/4/7/8/15/16*	–	12	18/18	36
3	difficult feature search – orientation	10	–	–	7/15/31	12	18/18	36
4	single feature search – colour	–	7/15/31	–	–	12	18/18	36
5	feature conjunction search	–	3/4/7/8/15/16*	3/4/7/8/15/16*	–	12	18/18	36
6	difficult feature search – orientation	–	–	–	7/15/31	12	18/18	36

Notes: In priming trials, the location of the target was identical to the one in the preceding trial. Priming trials were presented in sequences of three. This means that the same location was repeated three times. In standard trials, the location of the target item was different from the target location in the previous trials.* The two kinds of distractors were paired such that there was the same total number of distractors (set sizes: 7/15/31) as in the other two tasks.

Reaction (search) time (until button release), movement time (time between button release and target touch) and pointing error (distance between touch and target location in mm) were computed (see Table 3). Furthermore, the percentage difference between the reaction time for the first and third presentation of priming trials was computed as an indicator of implicit location learning. The assumption being that when participants remember the location of the previous trials, they should take less time to detect the target, thus leading to shorter reaction time for the third repetition and thus a bigger difference between reaction times for the first and third presentation of the same target location.

**Table 3:** Group means (±standard deviations) and group differences in the standard tests.

Test	MS patients	Healthy individuals	*p*
SPART/SPARTDR	18.57 (±5.39)/6.51 (±2.50)	20.05(±6.04)/7.08 (±2.57)	.144/.335
PASAT-3’’	44.69 (±10.09)	49.73 (±9.44)	.028
Digit span forward/backward	7.76 (±1.88)/6.71 (±1.93)	8.32 (±1.9)/8.18 (±1.91)	.194/.001
Spatial span forward/backward	8.34 (±2.27)/7.76 (±1.82)	8.57 (±1.99)/8.4 (±1.71)	.630/.115
Logical Memory I	25.63 (±8.98)	29.68 (±6.28)	.023
Go/Nogo; reaction time/errors	572.3 (±85.13)/1.1 (±2.36)	541.75 (±73.68)/0.48 (±1.24)	.094/.158
Divided attention; omissions	3.5 (±4.80)	1.08 (±1.4)	.005

Notes: SD: standard deviation; SPART: 10/36 Spatial Recall Test; SPARTDR: 10/36 Spatial Recall Test delayed recall; PASAT-3’’: Paced Auditory Serial Addition Test-3 seconds.

### Procedure

All participants were tested separately in a quiet room. The tests were conducted in the following order:

1. SPART
2. Go/Nogo
3. Divided attention
4. SPART delayed recall
5. Digit span
6. Spatial span
7. Logical Memory I
8. PASAT-3’’
9. Visual search

Twenty-eight participants (seven males, 21 females) of the healthy control group with a mean age of 35.6 years (SD: 11.49) were re-tested with the same tasks three to six months later (mean: 3.56; SD: 0.79), in order to check for potential practice effects. When parallel versions were available, the parallel test versions were used for the second assessment (SPART, pattern 2; PASAT-3’’, version B). In the case of Logical Memory I, we created a parallel version by using the first story for the first assessment and the second story for the second test session. 

### Statistics

Mixed 2x3 ANOVAs with the between factor “group” (MS, healthy) and the within factor “visual search task” (single feature search – colour, feature conjunction search, difficult feature search – orientation) for mean reaction time and movement time were conducted. Independent sample *t* tests were computed to assess group differences. Paired-samples *t* tests were performed to determine potential practice effects. Furthermore, a receiver operating characteristic (ROC) analysis was conducted, including the tests for which significant group differences were detected. Spearman’s correlation was used to determine the relationship between the measures of visual search and EDSS score, visual function score within the EDSS and time since diagnosis in MS patients respectively. The alpha level was set at *p*=.05 for all analyses, which were computed with IBM SPSS Statistics 19.

## Results

### Neuropsychological standard tests

There were significant differences between MS patients and healthy individuals in the PASAT-3’’, Digit span backward, Logical Memory I, and Divided attention test, with MS patients generally showing inferior performance (see Table 3). No significant group differences were found for the remaining tests.

### Visual search task

There was a significant main effect of group (*F*(1,76) = 17.17; *p*<001) and visual search task (*F*(1.36,103.42) = 41.48, *p*<001), as well as a significant group x visual search task interaction (*F*(1.36,103.42) = 6.48, *p*<.05) for reaction time. MS patients showed a prolonged reaction time in all tasks compared to healthy individuals, and this difference was most pronounced in the feature conjunction search (single feature search – colour: MS: *M* = 844.94; SD = 426.73; healthy: *M* = 539.51; SD = 176.69; feature conjunction search: MS: *M* = 963.65; SD = 380.78; healthy: *M* = 696.68; SD = 208.14; difficult feature task – orientation: MS: *M* = 1190,26; SD = 697.59; healthy: *M* = 725.27; SD = 275.82).

As the discriminatory power between the three tasks were comparable (see ROC analysis) despite the interaction between group and visual search task, the following analyses were conducted using the combined data of the three visual search tasks.

The same was done for mean movement time. For movement time no significant group x visual search task interaction (*F*(2,152) = 0.261; *p*=.77) was found. MS patients also showed a significantly prolonged movement time compared to healthy individuals (see Table 4 for further details). There were no significant differences between groups regarding priming effects and spatial pointing error. No significant correlations were detected between EDSS score and reaction time or movement time, or time since diagnosis and reaction or movement time (all *p*’s>.05). Likewise, no significant correlations were found between the measures of visual search and the visual function score (all *p*’s>.05). There were no significant differences between patients with (n = 14) and patients without (n = 24) movement deficits of the right arm, neither for reaction time (*t* (36) = 0.075; *p*=.94) nor for movement time (*t* (36) = 0.616; *p*=.542) or movement accuracy (*t* (36) = 0.138; *p*=.891).

**Table 4:** Group means (±standard deviations) and group differences in the different parameter of the visual search task.

Function	Parameter	MS patients	Healthy individuals	*p*
Visual attention	Reaction time (RT) in ms (±SD)	870.06 (±393.80)	569.09 (±163.15)	<.001
Implicit learning	Percentage difference between RT of the 1st and 3rd presentation (priming) (±SD)	32.32 (±15.44)	27.79 (±15.54)	.202
Motor execution	Movement time in ms (±SD)	739.17 (±124.95)	615.35 (±113.89)	<.001
Movement accuracy	Spatial pointing error (distance between target location and endpoint of the pointing movement; ±SD)	10.02 (±1.86)	10.49 (±1.41)	.209

Notes: RT: reaction time; ms: milliseconds

### ROC analysis

**Figure 3:**
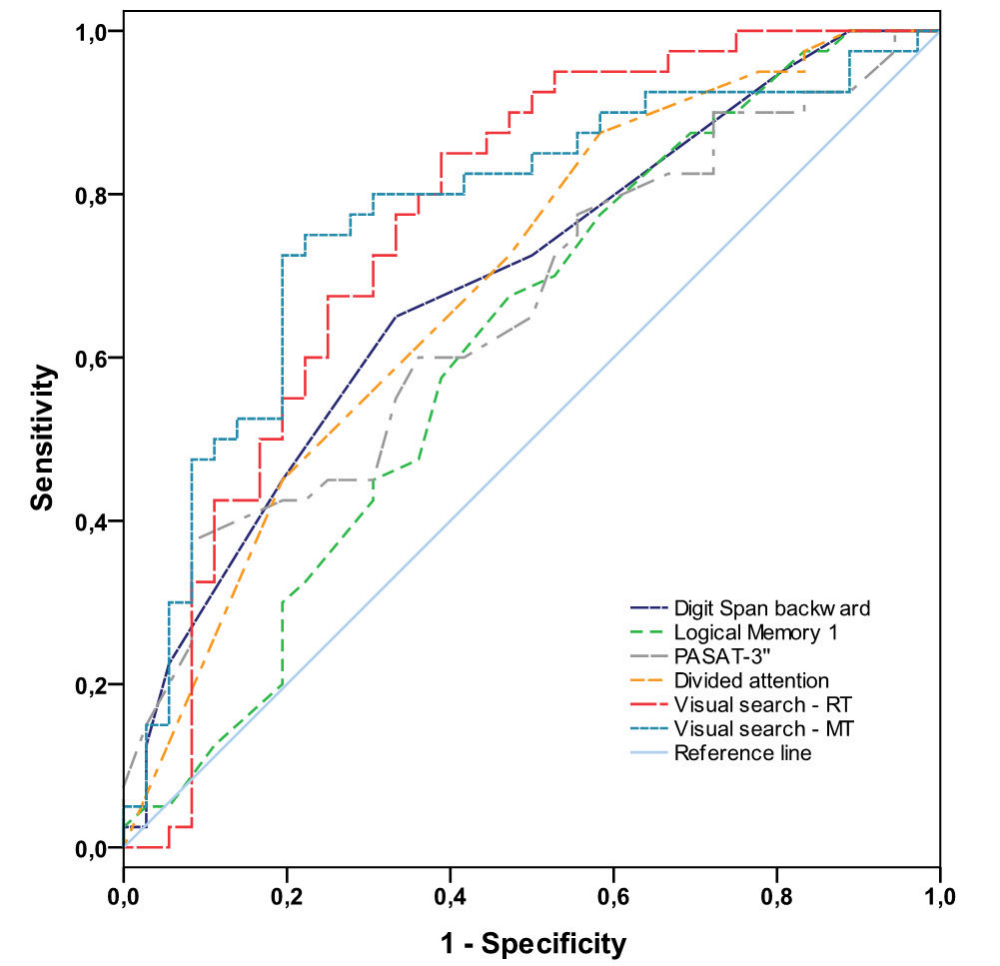
Receiver-Operator-Characteristic-Curves of the tests with significant group differences. Abbreviations: PASAT-3’’: Paced Auditory Serial Addition Test-3 seconds; RT: Reaction time; MT: Movement time. For reasons of clarity, only the curves of the combined mean RT and mean MT of all three visual search tasks are displayed.

Figure 3 illustrates the ROC-curves and Table 5 shows the respective Areas Under the Curve (AUC). Mean reaction time and movement time in the different visual search tasks, as well as for the combined data of all three tasks, turned out to discriminate best between healthy individuals and MS patients, as indicated by the highest AUC values. 

**Table 5:** Areas Under the Curve (±Standard Error of the Mean) of the neuropsychological tests.

Test	AUC (±SEM)	*p*
PASAT-3’’	.66 (±.06)	<.05
Digit span backward	.69 (±.06)	<.05
Logical Memory I	.62 (±.06)	<.05
Divided attention	.69 (±.06)	<.05
Singe feature search colour – RT	.75 (±.06)	<.001
Singe feature search colour – MT	.78 (±.05)	<.001
Feature conjunction search – RT	.74 (±.06)	<.001
Feature conjunction search – MT	.78 (±.05)	<.001
Difficult feature search – RT	.75 (±.06)	<.001
Difficult feature search – MT	.73 (±.06)	<.001
Visual search – RT (all 3 tasks)	.76 (±.06)	<.001
Visual search – MT (all 3 tasks)	.77 (±.06)	<.001

Notes: AUC: Area Under the Curve; SEM: Standard Error of the Mean; PASAT-3’’: Paced Auditory Serial Addition Test-3 seconds; RT: Reaction time; MT: Move time.

### Practice effects

There were significant differences between the first and second measurement in the re-tested healthy individuals for the PASAT-3’’ and Digit span forward with better performance during the second measurement (see Table 6). Marginally significant differences were found for SPART and divided attention. The performance in the remaining tests did not differ significantly between the two testing sessions. 

**Table 6:** Comparison of practice effects for different neuropsychological tests in a group of healthy participants (n=28).

Test	T1, mean (±SD)	T2, mean (±SD)	*p*
SPART/SPARTDR	22.14 (±4.94)/7.82 (±2.21)	24.11 (±3.58) /8.04 (±2.12)	.063/.665
PASAT-3’’	50.21 (±10.49)	52.57 (±9.85)	<.05
Digit span forward/backward	8.32 (±2.06)/8.39 (±2.04)	9.00 (±2.02)/7.86 (±2.03)	<.05/.229
Spatial span forward/backward	8.61 (±1.95)/8.61 (±1.57)	9.43 (±2.25)/8.43(±1.77)	.073/.525
Logical Memory I	30.71 (±6.29)	31.36 (±4.82)	.564
Go/Nogo; reaction time/errors	524.25 (±81.75)/0.39 (±1.34)	530.57 (±73.15)/0.14 (±0.36)	.685/.363
Divided attention; omissions	1.25 (±1.53)	0.79 (±1.34)	.056
Visual search –RT	559.42 (±156.29)	566.60 (±186.95)	.844
Visual search –MT	609.22 (±109.50)	600.93 (±154.71)	.808

Notes: The table presents the means (±standard deviations) for the two examination times and the p-value for the paired-sample t-tests, which was used to compare the performance between the two examination times. Abbreviations: SD: standard deviation; T: time of measurement; SPART: 10/36 Spatial Recall Test; SPARTDR: 10/36 Spatial Recall Test delayed recall; PASAT-3’’: Paced Auditory Serial Addition Test-3 seconds; RT: Reaction time; MT: Movement time.

## Discussion

We compared a selection of commonly used cognitive tests with the paradigm of visual search regarding their ability to discriminate between healthy individuals and MS patients and regarding their potential practice effects. Reaction time and movement time in the visual search task turned out to discriminate best between the two groups, without showing any practice effects, confirming the results of Ball et al.[13]. This suggests that the visual search task is a suitable method to assess cognitive impairment in MS patients and furthermore potentially disease- or medication- induced changes in MS patients due to robustness against practice effects.

Inferior mean performance in visual-attentional functions were observed in the visual search task for MS patients as indicated by longer reaction times for this group compared to the healthy individuals. However, no group differences regarding priming effects were found, meaning that implicit learning was unimpaired in our MS patients, as previously reported[1]. This is in line with findings in Alzheimer’s patients who also demonstrate intact perceptual priming[17] suggesting that implicit memory is a surprisingly robust skill that remains intact even when many other cognitive functions decline. 

The lack of group differences concerning reaction time between patients with and without movement deficits of the right arm, as well as the lack of correlation between reaction time and visual deficits, suggests that this outcome measure is relatively robust against mild motor and visual defects. 

Findings from our battery of standard neuropsychological tests mirrored those from previous studies: MS patients showed significantly lower performance in tests assessing verbal long-term memory[18], divided attention[3], information processing speed[2], as well as verbal short-term memory/working memory (digit span backward[19,20]), but not in the simpler version of this task (forward[2]). However, for all of those standard tests the power to discriminate between healthy and MS participants was inferior to that of the visual search task. Interestingly, even the most commonly used test, the PASAT, showed considerable lower discriminatory ability and was moreover prone to practice effects, providing further ammunition for those who are critical of the PASAT’s usefulness as a reliable measure of cognitive function[10]. 

Given the increasing recognition of cognitive impairment in MS, different strategies have been proposed for its assessment, such as test batteries like the BRB-N[21] or the Minimal Assessment of Cognitive Function in Multiple Sclerosis (MACFIMS)[22], patient- or family member-reported scales[23,24] and different screenings such as single tests[25], a collection of tests[26] or internet-based cognitive testing[27]. However, whereas test batteries are too time-consuming, self- or family member-reported scales are not reliable[4,6], and many screening tests assess only one or few, mostly speech-dependent aspects of cognition and are often susceptible to measurement errors and practice effects, thus being inappropriate for the assessment of changes in cognitive functions[10,28].

The administration duration of the visual search task was between 15 and 20 minutes. However, as there were no differences regarding the performance during the first three and the second three blocks, the task duration could be shortened to seven to 10 minutes, thus being shorter than most of the available screening procedures. In fact given that all three search tasks discriminated equally well between participants with and without MS, it would be feasible to restrict the examination to just one of the three search tasks, thereby reducing the examination time to approximately 5 minutes. 

Despite its brief administration duration, the visual search task assesses many different facets of cognitive functions, especially those frequently neglected by conventional declarative tests, such as visual exploration and visual memory. 

Correspondingly, Feinstein et al.[29] recently found that the phenomenon of inattentional blindness, whereby healthy individuals fail to notice a perfectly visible person/object when they are concentrating on another task, is observed less in MS patients. This is taken as a sign of increased distractibility in MS patients. Interestingly, this pervasive deficit is not picked up by the MACFIMS[22] battery: MS patients with abnormal performance in the inattentional blindness task showed normal performance in the MACFIMS tests. The authors conclude that this could explain why some MS patients have difficulties in daily life situations requiring the filtering of distractors or multitasking without showing deficits in test batteries[29]. The visual search task assesses distractibility quite explicitly since successful performance requires that participants focus on a given target feature while filtering out irrelevant but often salient distracting features. This aspect of the visual search paradigm may in part account for its superior power to discriminate between participants with and without MS. 

The visual search task offers other advantages which so far have not been mentioned. It is computer-based, therefore can be carried out by the patients without supervision and can also be automatically analyzed, thus yielding objective test results with minimal effort. 

Since the mean disease duration of our sample of MS patients was over seven years, we do not know whether the same results can be expected for a sample of early-stage MS patients. We should also note another limitation of our study. Our procedure to identify sensitive cognitive tests was based on a comparison between healthy participants and MS patients. Tests which discriminate reliably between MS patients and healthy adults will help in the diagnosis of MS patients but may not provide the best measures to examine the impact of clinical drugs on disease progression. To find out whether our visual search tasks provide the sensitivity to detect spontaneous or therapy-induced changes over time a longitudinal study will be needed. 

In conclusion, visual search seems to be a good tool for the assessment of cognitive impairments in MS due to its good discriminatory power, easy and objective computer-based administration, high accuracy of measurement due to the great number of trials, and assessment of a wide range of cognitive skills, which are often neglected by conventional procedures. Furthermore the visual search task is not susceptible to practice effects (at least in healthy individuals), predestinating this tool for the assessment of cognitive changes, i.e. due to disease progression or disease-modifying treatment.
